# The Gender Cliff in the Relative Contribution to the Household Income: Insights from Modelling Marriage Markets in 27 European Countries

**DOI:** 10.1007/s10680-019-09547-8

**Published:** 2020-01-10

**Authors:** André Grow, Jan Van Bavel

**Affiliations:** 1 Laboratory of Digital and Computational Demography, Max Planck Institute for Demographic Research, Konrad-Zuse-Straße 1, 18057 Rostock, Germany; 2 Centre for Sociological Research, University of Leuven (KU Leuven), Parkstraat 45, 3000 Leuven, Belgium

**Keywords:** Marriage markets, Income, Gender norms, Gender inequality, Simulation modelling

## Abstract

In Western countries, the distribution of relative incomes within marriages tends to be skewed in a remarkable way. Husbands usually do not only earn more than their female partners, but there is also a striking discontinuity in their relative contributions to the household income at the 50/50 point: many wives contribute just a bit less than or as much as their husbands, but few contribute more. This ‘cliff’ has been interpreted as evidence that men and women avoid situations where a wife would earn more than her husband, since this would go against traditional gender norms. In this paper, we use a simulation approach to model marriage markets and demonstrate that a cliff in the relative income distribution can also emerge without such avoidance. We feed our simulations with income data from 27 European countries. Results show that a cliff can emerge from inequalities in men’s and women’s average incomes, even if they do not attach special meaning to a situation in which a wife earns more than her husband.

## Introduction

Women’s labour market opportunities have improved dramatically since the mid of the twentieth century. Starting in the 1960s, women have entered higher education in ever greater numbers and nowadays they often outperform men in terms of enrolment and success in tertiary education (Blossfeld and Buchholz 2009; DiPrete and Buchmann 2013; Van Bavel et al. 2018). This was paralleled by an increase in female labour force participation and an influx of women into previously male-dominated occupations of high status (Baxter et al. 2005; Cha 2013; Goldin 2014; Ruggles 2015). Because of these changes, some scholars have expected marriages to become increasingly gender egalitarian, meaning that spouses would become more and more similar in their labour force participation and contributions to the economic well-being of their families (e.g., Jalovaara 2012; Nock 2001; Oppenheimer 1988; Torr 2011).

There is evidence that the economic roles that men and women play within their families have indeed become more similar over time (Blossfeld and Buchholz 2009; Goldscheider et al. 2015; Sweeney and Cancian 2004; Torr 2011). However, marked gender differences still exist in many areas of life (Bailey and DiPrete 2016; England 2010; Esping-Andersen 2009; Goldin 2014; Ridgeway 2011), also when it comes to the financial contributions that men and women make to their families (Bertrand et al. 2015; Brennan et al. 2001; Klesment and Van Bavel 2017; Rogers and DeBoer 2001; Van Bavel and Klesment 2017). Across households, men not only provide a larger share of the household income, but there often is also a remarkable discontinuity at the 50/50 point.

**Fig. 1:**
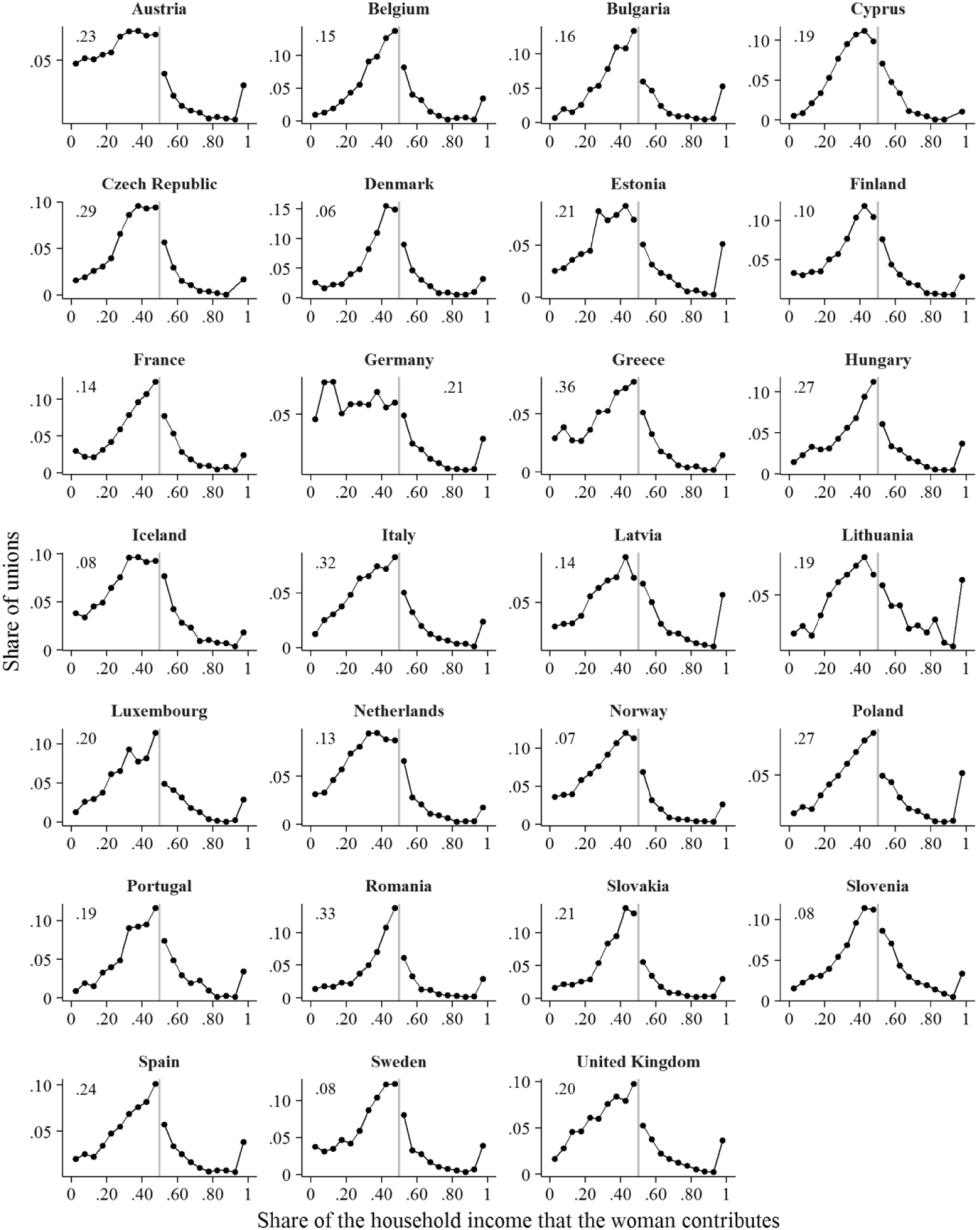
Shares that women contribute to household income in 27 countries. *Note*: The grey vertical line indicates the point where the share of the household income that the woman provides is .5. The number in the upper left/right corner of each panel shows the share of couples in which the woman contributes nothing to the household income. *Source*: Pooled data from the 2007 and 2011 waves of the cross-sectional versions of EU-SILC

Figure 1 illustrates this based on data from the European Union Statistics on Income and Living Conditions (EU-SILC). The selected sample contains unions (living in marriage or unmarried cohabitation) among people age 25–45 years in 27 European countries, observed in 2007 and 2011.^1^ The figure plots the distribution of couples according to the share of the household income that the woman contributes (see detailed data description below). As can be seen from Fig. 1, in most countries, the relative income distribution steeply increases from the point where the woman contributes nothing to the household income, up to the point where she contributes close to 50%. After this point, there typically is a sharp drop and there are much fewer unions in which the woman contributes more than the man.

One interpretation in the literature holds that this ‘cliff’ in the relative income distribution results from persistent gender norms that favour the traditional male breadwinner/female homemaker model. More specifically, the fact that there is a discontinuity at the 50/50 point may indicate a social norm that ‘a man should earn more than his wife’ (Bertrand et al. 2015, p. 612).

The observed discontinuity in the distribution of relative incomes within households would be consistent with a norm that favours male superiority in income, if such a norm existed. However, in this paper, we argue that such a norm is not necessary to generate a discontinuity. Instead, we suggest that a cliff may emerge even if both men and women prefer partners with high income over partners with low income, if we consider that even in the most gender egalitarian societies women’s average income is lower than men’s.

Our argument is based on the following intuition. If people strive for high-income partners, men who rank high in the male income distribution will be in the best position to compete for women who rank high in the female income distribution, vice versa. Some men may therefore form unions with similar-income partners, but because women’s average income is lower, many men will face a shortage of partners with similar or even higher income. Unless they are willing to remain single, these men will have to form unions with women who earn less than they do. Women, by contrast, will have to ‘settle’ less often for a lower-income partner. These differences in men’s and women’s marriage market opportunities are likely to not only create a right skew in the distribution of women’s contribution to household income, but also a discontinuity close to the 50/50 point. This occurs even if people are not more aversive of a situation in which the wife out-earns her husband than of a situation in which he out-earns her.

We demonstrate the logical consistency and empirical plausibility of our argument with a simulation study in which we compare the outcomes of a simple marriage market model with the observed distributions of relative income in the 27 countries shown in Fig. 1. The model assumes that men and women strive for a high joint income in the unions that they form, while using their own income as a point of reference for determining the minimum income they expect in a partner. However, they do not evaluate a situation in which a wife out-earns her husband any differently from a situation in which he out-earns her. Our results show that partner choice based on this preference tends to generate a right skew in the distribution of relative incomes within households and, most importantly, a discontinuity at the 50/50 point.

In what follows, we present the model in some detail, first providing some theoretical background, then describing the algorithm that we employ in modelling the partner search process and discussing the empirical data that we use to create plausible marriage markets. We then submit the model to systematic simulation experiments, present our results, and close with discussing the implications that our findings have for current research practice and future research. We have implemented the model in the simulation modelling environment NetLogo (Wilensky 1999). The code can be obtained from [https://www.comses.net/codebases/4e97fa4a-db5b-4cff-acea-5314201af5ce/], together with a more technical model description and all scripts that are necessary to replicate our results.

## Modelling Marriage Markets

### Background

Observed heterosexual marriage patterns are commonly assumed to derive from two-sided partner search in a marriage market (Schwartz 2013; Willekens 2010). This notion holds that both men and women are searching for a spouse among the available alternatives of the opposite sex. Their search is guided by a set of preferences for the characteristics that their partner should have, but the realization of these preferences is constrained by the composition of the marriage market. If there is a shortage of alternatives with the desired characteristics, the opportunities to find the ‘ideal’ mate are limited, and people somehow need to adjust to this reality. These adjustments can take different forms, such as widening and prolonging search, settling for a partner who is less than ideal, or even foregoing marriage altogether (England and Farkas 1986; Oppenheimer 1988).

Past research suggests that men and women somewhat differ in the characteristics they prefer in a partner, so that women tend to place more emphasis on the economic prospects and status of potential partners than men (Buss 1989; Buss et al. 1990). Sociological explanations of this difference suggest that it reflects traditional differences in women’s and men’s roles in society (Eagly et al. 2009). In the past, women’s role was traditionally located in the home, and even if they worked, they tended to be overrepresented in low-paying occupations. This made their economic well-being largely dependent on the income of their husbands, which explains why they used to put greater emphasis on income in their partners than men. This difference was also reflected in societal gender norms, that held that a man should be the main provider for his family. A situation in which a husband was out-earned by his wife was therefore potentially threatening for his male gender identity (Bertrand et al. 2015; Schwartz and Han 2014). Yet, with the increasing convergence in men’s and women’s economic roles that has taken place since the mid of the twentieth century, this situation has changed, particularly in the Western world. That is, men’s and women’s partner preferences have become more similar (Zentner and Eagly 2015; Zentner and Mitura 2012) and people’s aversiveness to not complying with the traditional male breadwinner family model has decreased (Meeussen et al. 2018).

While there are still differences in men’s and women’s partner preferences, and while traditional gender norms may persist in some countries and some parts of the population, our goal here is to conduct a ‘what-if’ experiment of the following type: What if men’s and women’s partner preferences were the same and there was no norm that holds that a husband should earn more than his wife? In such a situation, would the existing income differences between them be enough to generate a cliff in the relative income distribution as shown in Fig. 1? Hence, from here on we assume that men and women put the same emphasis on the income of potential partners, and search for a partner with high income, without attaching special meaning to a situation in which the woman out-earns her partner. Next to this, we need to make some additional assumptions as to how people go about their search for a partner, to conduct our experiment.

One of the most important features of the partner search process is the fact that the information that people have about the available alternatives is usually incomplete. This creates the problem of trading off the utility from marrying one of the currently available alternatives against the utility from possibly finding somebody even more attractive in the future, net of the costs that prolonged search creates. Perfectly rational actors with unlimited cognitive capabilities can solve this problem by calculating their optimal reservation quality, which defines the minimal quality they should strive for in the characteristics of their partner to balance their expected search costs, given the available information about the composition of the marriage market. They should stop their search when they find a partner whose characteristics are equal to or exceed this quality (Batabyal 2009; Keeley 1977; Mortensen 1988). Yet, as Todd and colleagues (Todd 1997; Todd et al. 2005, 2013; Todd and Miller 1999) have highlighted, the calculations that are necessary for this are often too complex to be applied by the average person in real life. Instead, people tend to apply decision heuristics that are much simpler but still yield satisfactory outcomes.

One heuristic that has received much attention in earlier research is using the quality of one’s own characteristics as a point of reference for selecting a partner (e.g., Kenrick et al. 1993; Kirkpatrick and Ellis 2001, 2006; Penke et al. 2007; Regan 1998; Skopek et al. 2011; Sloman and Sloman 1988; Todd et al. 2007, 2013). This heuristic tends to be efficient, because people who have high-quality characteristics are in high demand and have good chances of attracting partners who also have high-quality characteristics. They can therefore afford to set their aspirations high and still find a partner with reasonable search effort. People with characteristics that are in lower demand, by contrast, are likely to experience more difficulties in attracting partners with high-quality characteristics. They therefore often need to set their aspirations lower, to avoid engaging in excessive search efforts that do not necessarily lead to marriage (Penke et al. 2007).

As Kalmijn (1998) highlighted, socioeconomic resources are among most the important characteristics that people consider in potential partners. The reason is that ‘[e]conomic well-being is shared by the family members […]. As a result, the income and status of one spouse contribute to the income and status of the other by raising the income and status of the family’ (Kalmijn 1998, p. 399). If we assume that high income is indeed an important characteristic in partnering decisions for both men and women, the above heuristic implies that people should strive for partners who earn at least as much as, or more than, they do. They should be more reluctant to partner with somebody who earns less. In this way, they minimize the risk of settling for a partner whose income is too low, considering their own attractiveness on the marriage market.

### The Model

Our model implements the principles discussed in the previous section in the following way. First, we assume that income is the only characteristic that people consider in their partnering decisions and that both men and women strive for partners with high income. Second, we assume that people’s information about the composition of the marriage market (i.e., about the incomes of the available opposite-sex members) is incomplete. They therefore must sample the available alternatives one-by-one to find a partner. This sampling takes the form of random meetings with opposite-sex members, during which they learn about each other’s income. Third, to avoid settling for a partner ‘too early’, they engage in a prolonged courtship period before fully committing to any of the available alternatives. Courtship is here represented by dates that after some time may turn into marriage. During the courtship period, people continue sampling from the available alternatives, and they may leave their current partner when they meet a person whose income is higher than that of their current partner and who is also willing to accept them as a partner (see Simão and Todd 2002 for a similar approach to modelling partner search).

Furthermore, and most importantly, we assume that for people who are dating somebody, their decision to stop their search and settle for their current partner is guided by their income relatively to that of their partner, as well as by the time they have been looking for a better alternative without success. In more detail, we assume that people are more likely to settle for a partner who earns at least as much as they do, than they are to settle for a partner who earns less than they do. Yet, they become increasingly willing to settle for *any* partner, the longer they have already been looking for a better alternative without success. When two dating individuals both decide to stop their search and settle for their current partner, they get married and are permanently removed from the marriage market.

More technically, the model is a two-sex model that consists of a closed population of male and female individuals, which are indexed by *i*.^2^ Next to their gender (*g*_*i*_), individuals can be described by their annual income (*y*_*i*_) and their relationship status (*r*_*i*_). Income is fixed and assigned probabilistically at the beginning of a given simulation run, based on the empirically observed income distributions that we describe in the next section. Individuals’ relationship status can take the states ‘single’, ‘dating’, or ‘married’, and this state can change over the course of a simulation run.

Each simulation proceeds in discrete time steps, index by *t*. In each time step, people’s search for a partner takes the form of random meetings with opposite-sex members. The model assumes that single individuals are always looking for a partner and therefore are always available for meeting opposite-sex members. By contrast, individuals who are currently dating are only available for meetings if they have not stopped their search yet (see details below). Married individuals are not on the marriage market and therefore do not take part in meetings.

Whenever two individuals meet, they need to decide whether they want to start dating each other and leave potential current partners for this. Income is pivotal in these decisions. Single individuals are always willing to date the person they have just met, regardless of their income.^3^ Yet, those who are already dating somebody else are only willing to leave their current partner and start dating the person they have just met, when the income of this alternative is higher than the income of their current partner. Two individuals actually start dating if they are *both* willing to do so. Hence, even if a given individual *i* is willing to date *j*, this will not happen unless *j* is also willing to date *i*.

**Fig. 2:**
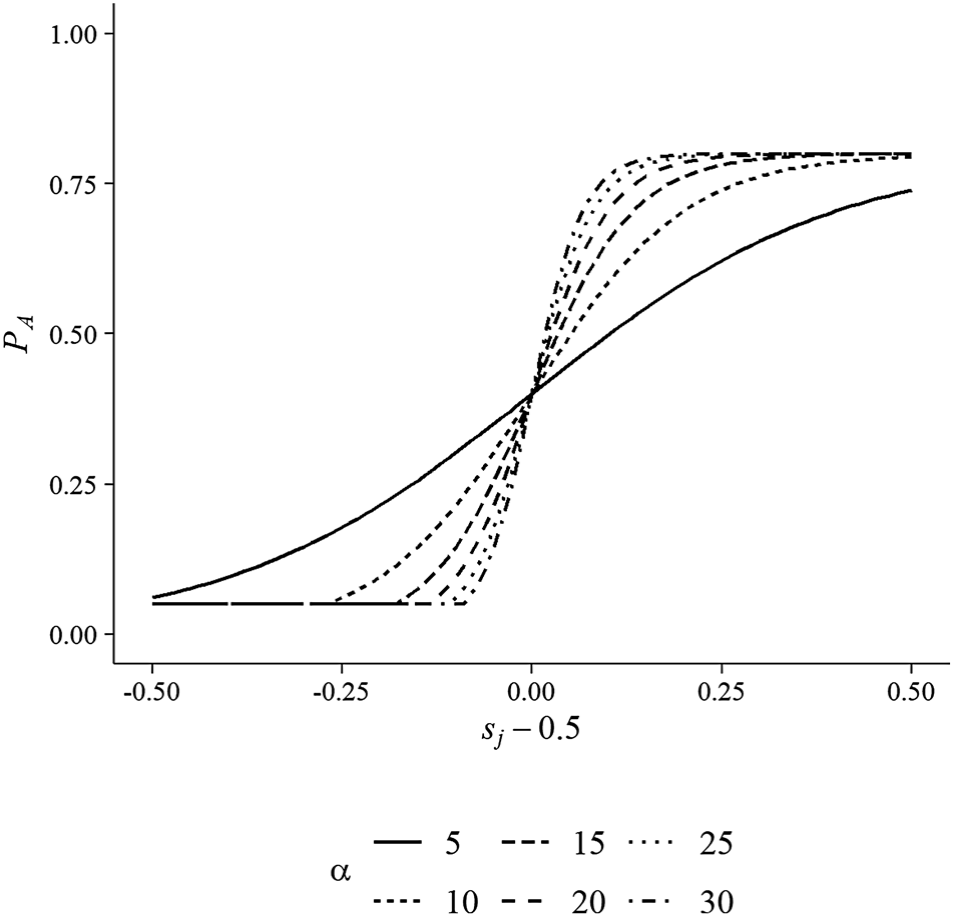
Baseline probability that individual *i* is willing to settle for his/her partner *j*, contingent on how much the share of the household income that *j* would contribute deviates from .5 (*s*_*j*_ − .5) at different levels of *α*

As indicated above, individuals who are dating somebody might decide to stop their search and settle for their current partner. These decisions are made probabilistically at the beginning of each time step. The probability that a given dating individual *i* will cease his/her search depends on his/her partner’s income relatively to *i*’s and on the time that *i* has spent already looking for a better alternative without success. Formally, the baseline probability that *i* is willing to stop searching and settle for his/her partner *j* is defined as

1 $$ P_{A} = m\frac{{{\text{e}}^{{\left( {s_{j} - .5} \right)\alpha }} }}{{1 + {\text{e}}^{{\left( {s_{j} - .5} \right)\alpha }} }}, $$

where *s*_*j*_ is the share that *i*’s current partner *j* would contribute to the overall household income if they would get married (calculated as *s*_*j*_ = *y*_*j*_/(*y*_*j*_ + *y*_*i*_)), and the parameter *m* limits the maximal value of $P_{A}$. Setting *m* < 1 ensures that even individuals who have already found a partner who earns much more than they do invest at least some effort in finding a partner with even more income. Furthermore, we set the lower limit of $P_{A}$ to 0.05, so that even individuals who do not contribute anything to the household income (i.e., who have no income) have some chance of getting married (*s*_*j*_ is set to .5 if both partners have no income). The parameter *α* governs how much individuals differentiate between potential spouses who earn less than they do and spouses who earn at least as much as they do. Figure 2 illustrates this for different *α*-values, at all possible values of *s*_*j*_, with *m* = .8. As the figure shows, as *α* becomes larger, the baseline probability that *i* is willing to cease his/her search is much lower when *j* earns less than *i* (i.e., when *s*_*j*_ − 0.5 < 0) than when *j* earns as much as, or more than, *i* (i.e., when *s*_*j*_ − 0.5 ≥ 0).

Even though our artificial individuals are aversive of accepting somebody who makes less than they do as partner, this aversion decreases when they fail to find a better alternative who is also willing to accept them as a partner. We implement this by weighting $P_{A}$ by the number of time steps that the focal individual *i* has been looking for a better alternative without success at the beginning of time step *t* (*c*_*i,t*_):

2 $$ P_{B,t} = 1 - {\text{e}}^{{ - (P_{A} c_{i,t} )}} . $$

Equation (2) holds that individual *i* becomes increasingly willing to settle for their current partner, the longer he/she has been searching for an alternative already (i.e., the larger *c*_*i,t*_ becomes). Thus, individuals are striving for high-income partners and try to attain their goal by ‘trading up’ their current dates whenever they get the opportunity to do so. Yet, if they fail to find somebody better for some time, they become increasingly likely to settle for their current partner. This is particularly likely for those whose partners earn at least as much as they do.

Individuals who have stopped their search and settled for their partners become willing to marry them. They are therefore not available for meetings with opposite-sex members anymore. This does not mean that individual *i* automatically gets married the moment he/she ceases his/her search. Marriage happens only when his/her partner *j* also decides to stop his/her search. Before this happens, *j* may continue to meet opposite-sex members, and this may lead *j* to break up with *i*, if *j* encounters somebody who earns more than *i* and is also willing to date *j*. If this happens, *i* becomes single again and therefore is available for meeting with opposite-sex members again. However, the moment *j* also stops searching and settles for *i*, the two get married and are permanently removed from the marriage market.

To summarize, the processes that take place in each time step are as follows:

1. Determine for each man and woman whether they are currently searching a partner. Single individuals are always searching for a partner, but people who are currently dating may cease their search (based on the probabilities determined by Eqs. (1) and (2)). People who have ceased their search will remain in this state either (a) until the person they are dating also ceases their search or (b) until they lose the person they were dating to somebody else.
2. For all dates in which both individuals have ceased their search, let the involved individuals get married and remove them from the marriage market.
3. Each man who is looking for a partner is randomly paired with one woman who is also looking for a partner. At this moment, both need to decide whether they want to start dating the person they have just met. For people who are currently single, this is always the case. For people who are already dating somebody else, this is the case if the income of the person they have just met is higher than the income of their current date. When both want to date, they leave possible current partners and actually start dating.
4. Among all people who are dating, increase the number of time steps they are already looking for a better alternative by one.

These steps are iterated until all men and women have married.

### Empirical data

Similar to Klesment and Van Bavel (Klesment and Van Bavel 2017; Van Bavel and Klesment 2017), we used data from the 2007 and 2011 rounds of the EU-SILC. The EU-SILC is an annual cross-national survey that started with 15 participating countries in 2004. By 2007/2011, this number had increased to 31/32 countries, respectively. The survey employs a rotating panel, in which each subsequent wave replaces part of the sample, so that the entire sample is renewed every four years. We used the cross-sectional version of the survey, and the income reference years for the 2007/2011 waves are the years 2006/2010, respectively. For comparability, we focused on the same 27 countries as Klesment and Van Bavel (Klesment and Van Bavel 2017; Van Bavel and Klesment 2017). We weighted the data with the sampling weights that are provided with the EU-SILC and used the resulting data in two parts of our study.

First, we used the data for calculating the distributions of relative income shown in Fig. 1, which are the target of our simulation experiments. In doing so, we applied similar rules for case selection as Klesment and Van Bavel (2017). That is, we selected women who were living with a partner at the time of the survey (either in marriage or in unmarried cohabitation), who were between 25 and 45 years old, and whose partner was in the same age range.^4^ For calculating the share of the couple’s joint household income that the woman provides (*s*_*f*_), we focused on both partner’s annual gross income from paid employment and self-employment, only including couples in which at least one partner had positive income. This share was calculated as *s*_*f*_ = *y*_*f*_/(*y*_*f*_ + *y*_*m*_), where *y*_*f*_ and *y*_*m*_ refer to the woman’s and her partner’s income, respectively. Table 1 (Sample A) shows the number of unions that were included in the analysis, and Fig. 1 above shows the distributions of relative income within the selected unions. Note that women are more likely than men to have no income at all. There was thus a large share of couples in which the woman contributed nothing to the household income. To avoid that this large share affects the scaling of the figures when showing the relative income distributions across countries, we followed Klesment and Van Bavel (2017) and opted for displaying the share of this unions type as a number in the upper left/right corner of Fig. 1.

**Table 1:** Number of cases per country by sample

Country	Sample A: unions	Sample B: individuals	
		Women	Men
	*N*	*N*	*N*
Austria	2573	4591	4152
Belgium	2436	4278	4069
Bulgaria	1947	3712	3750
Cyprus	1649	3201	2668
Czech Republic	3534	6005	5869
Denmark	2421	3680	3214
Estonia	2052	3584	3400
Finland	4095	6050	6100
France	4354	7032	6621
Germany	4448	8274	6960
Greece	1074	2149	2061
Hungary	3973	7308	6905
Iceland	1541	2362	2317
Italy	7151	14,916	14,051
Latvia	1601	3429	3036
Lithuania	1590	2949	2664
Luxembourg	2634	4176	3990
Netherlands	4896	7292	6730
Norway	2389	3704	3566
Poland	5900	10,090	9370
Portugal	1785	3351	3190
Romania	3015	5148	5051
Slovakia	2256	4353	4179
Slovenia	4195	8412	8503
Spain	5453	10,459	9827
Sweden	2982	4586	4370
United Kingdom	3087	5597	4901

**Fig. 3:**
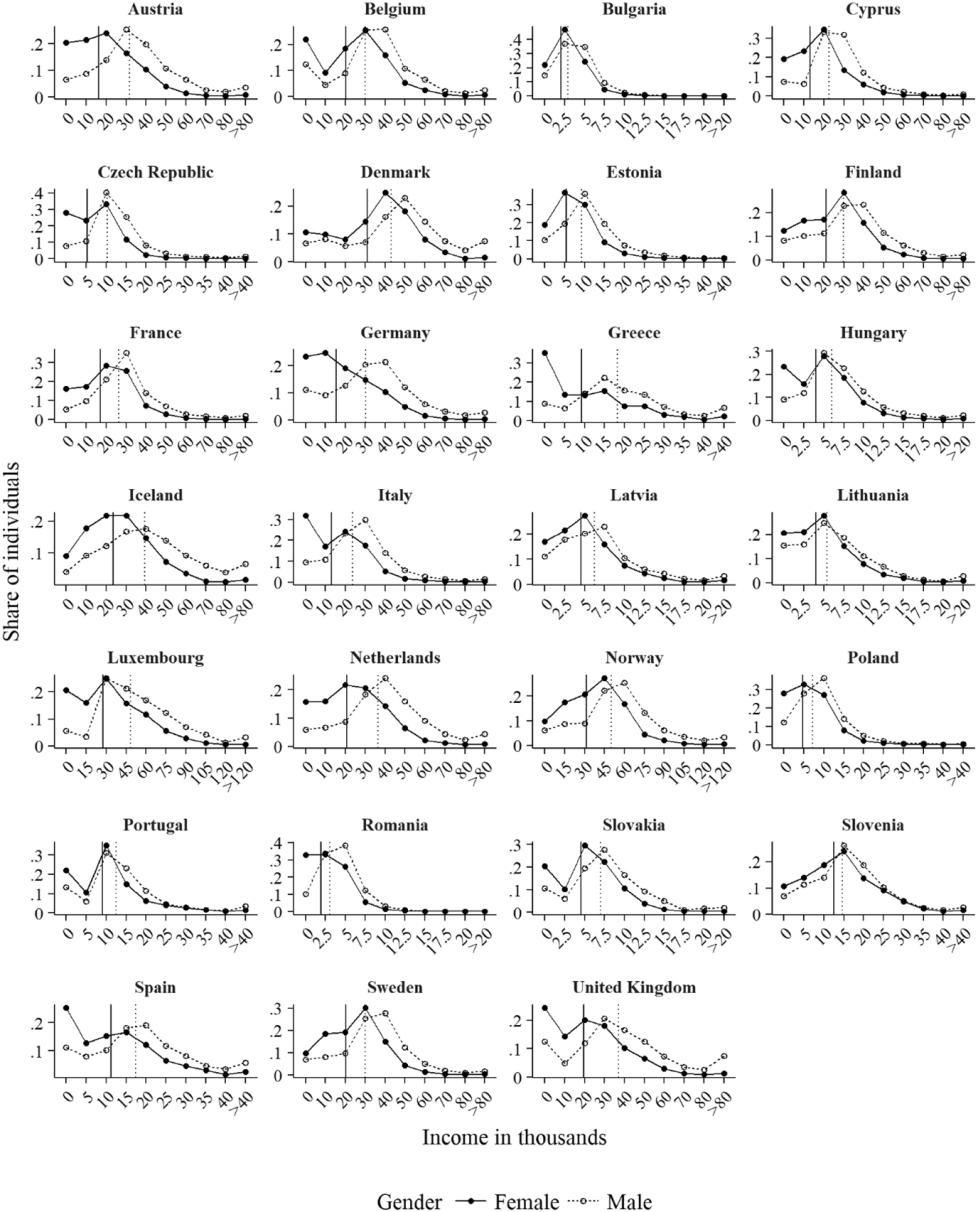
Comparison of women’s and men’s incomes across 27 countries. *Note:* Income is measured in national currencies. The vertical lines show the average incomes of men and women. *Source*: Pooled data from the 2007 and 2011 waves of the cross-sectional versions of EU-SILC

Second, we used the EU-SILC data for initializing the marriage markets in our simulation experiments in terms of the income distributions in each of the 27 countries that we considered. For this, we included all individuals between 25 and 45 years of age, regardless of their union status at the time of the survey (i.e., including individuals who were single, widowed, separated, married, or living in unmarried cohabitation). With this approach, we take the observed incomes of men and women as given and explore what the resulting relative income distributions would look like if members of both sexes would select their partners based on these incomes. Table 1 (Sample B) shows the case numbers per gender that we obtained and Fig. 3 shows the resulting income distributions.^5^ Across all 27 countries, women’s average income was lower than men’s, and women’s income distribution tended to be heavier on the left-hand side than men’s. This means that typically there were more women than men who earned comparatively little. At the same time, there were often many more men than women in the highest income categories.

### Experimental Setup

In our experiments, we assumed a population of 1000 men and 1000 women. These individuals were assigned their income probabilistically based on the country- and gender-specific income data that we obtained from the EU-SILC. To implement the notion that people use their own income as a point of reference when selecting a partner, we set *α* to 30, so that they strongly differentiated in their marriage decisions between those opposite-sex members who earn less than they do and those who earn as much as, or more than, they do. To assess how sensitive our results are to this assumption, we conducted an additional simulation experiment in which we explored the behaviour of the model for smaller *α*-values (i.e., *α* = 5, *α* = 10, *α* = 15, *α* = 20, and *α* = 25). For this, we focused on four countries that showed a large cliff, both in the empirical data and in the outcomes of our main simulation experiment (Belgium, Germany, Sweden, and the United Kingdom). Across simulation runs, we assumed that the maximum baseline probability for accepting somebody for marriage was 0.8 (i.e., *m* = 0.8). As indicated above, the lower limit of this probability was 0.05.

The outcome that we are interested in is the share of the income that the female members of our artificial unions contribute to the overall household income. As for the empirical data, we calculated this share as *s*_*f*_ = *y*_*f*_/(*y*_*f*_ + *y*_*m*_), where *y*_*f*_ and *y*_*m*_ are the individual incomes of the female and male partner, respectively.

Given the stochastic nature of the simulation process, we conducted 50 simulation runs per country and condition and averaged the outcomes across runs.

## Results

**Fig. 4:**
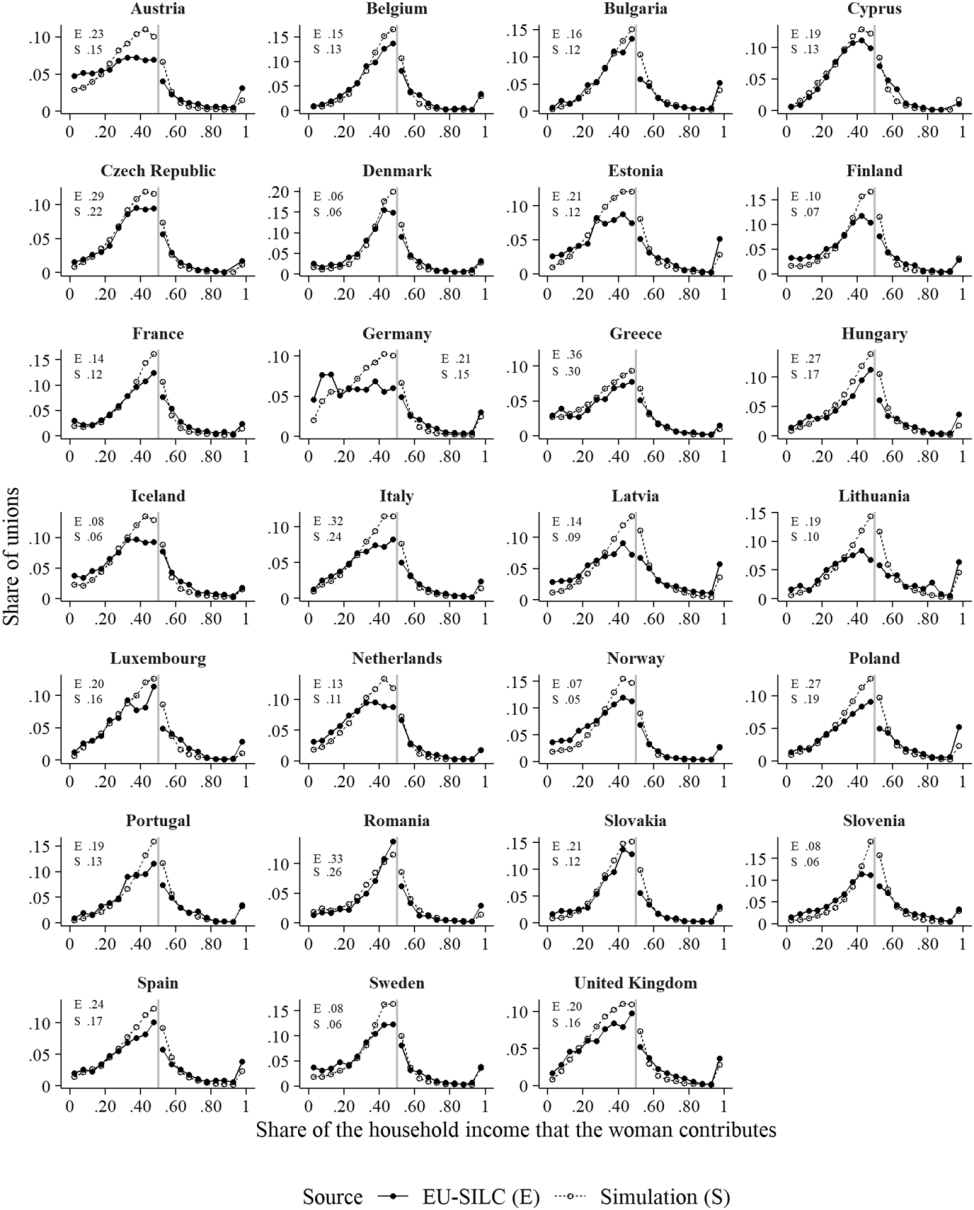
Comparison of the relative income distributions observed in the EU-SILC data with those generated by the simulation model. *Note:* The grey vertical line indicates the point where the share of the household income that the woman provides is .5. The numbers in the upper left/right corner of each panel show the shares of couples in which the woman contributes nothing to the household income. *Source*: The simulation results are based on the main simulation experiment. The empirical data are the same as for Fig. 1

Figure 4 shows the distributions of relative income that we obtained from our main simulation experiment and compares them with the distributions observed in the empirical data. As in Fig. 1, we show the share of couples in which the woman contributes nothing to the total household income in the upper left/right corner, distinguishing the empirical and the simulation data with ‘E’ and ‘S’, respectively. On average, it took about 36 time steps (SD = 4.3) for everybody to get married (at which point a given simulation run stopped).

The most striking, and most important, result that Fig. 4 illustrates is that in many of the countries that we consider here, the simulation model generated a cliff in the relative income distribution. This cliff was often similar to, but frequently was even stronger than, the cliff in the empirical data. The example of Sweden illustrates this nicely. In the empirical data, the last category before the 50/50 demarcation line (i.e., the category in which women earn about as much as men) contains about 12% of all couples, whereas the first category immediately after this line contains only 8% of all couples. In the simulation data, by contrast, these categories contain about 16% and 10% of all couples, respectively. Hence, the drop that the simulation model generates is two percentage points larger than the drop that we observed in the empirical data.

A second striking feature of the simulated distributions is that they tend to closely trace the empirical distributions at the low and high ends of the relative income distribution. That is, while the model tends to generate more couples with roughly similar income than observed empirically, it quite accurately captures the shares of couples in which women contribute very little or very much to the household income. This also applies to the most extreme categories, in which women contribute nothing to the household income, or in which their income accounts for almost the entire household income. The case of Belgium illustrates this well. In the EU-SILC data, women have no income in about 15% of all couples, whereas this value is 13% in the simulation results. Similarly, in both the empirical data and the simulation data, couples in which women’s income accounts for almost the entire household income make for about 3% of all couples.

In some countries, there are notable deviations from these general patterns. First, in the empirical distributions, the couple shares steeply increase in most countries from the point where women earn nothing, to the point where they earn almost as much as their partners. The only exceptions from this are Austria and Germany, where there are almost constant shares of couples in which the woman earns less than her partner, regardless of her/his exact income. The simulation model does not capture this well. Furthermore, the model does not generate a cliff in all countries. In particular, in Lithuania and Slovenia, the relative income distribution that the model generates is rather symmetric around the 50/50 demarcation line. Notably, in these countries, there also is no clear discontinuity at the 50/50 demarcation line in the empirical data.

**Fig. 5:**
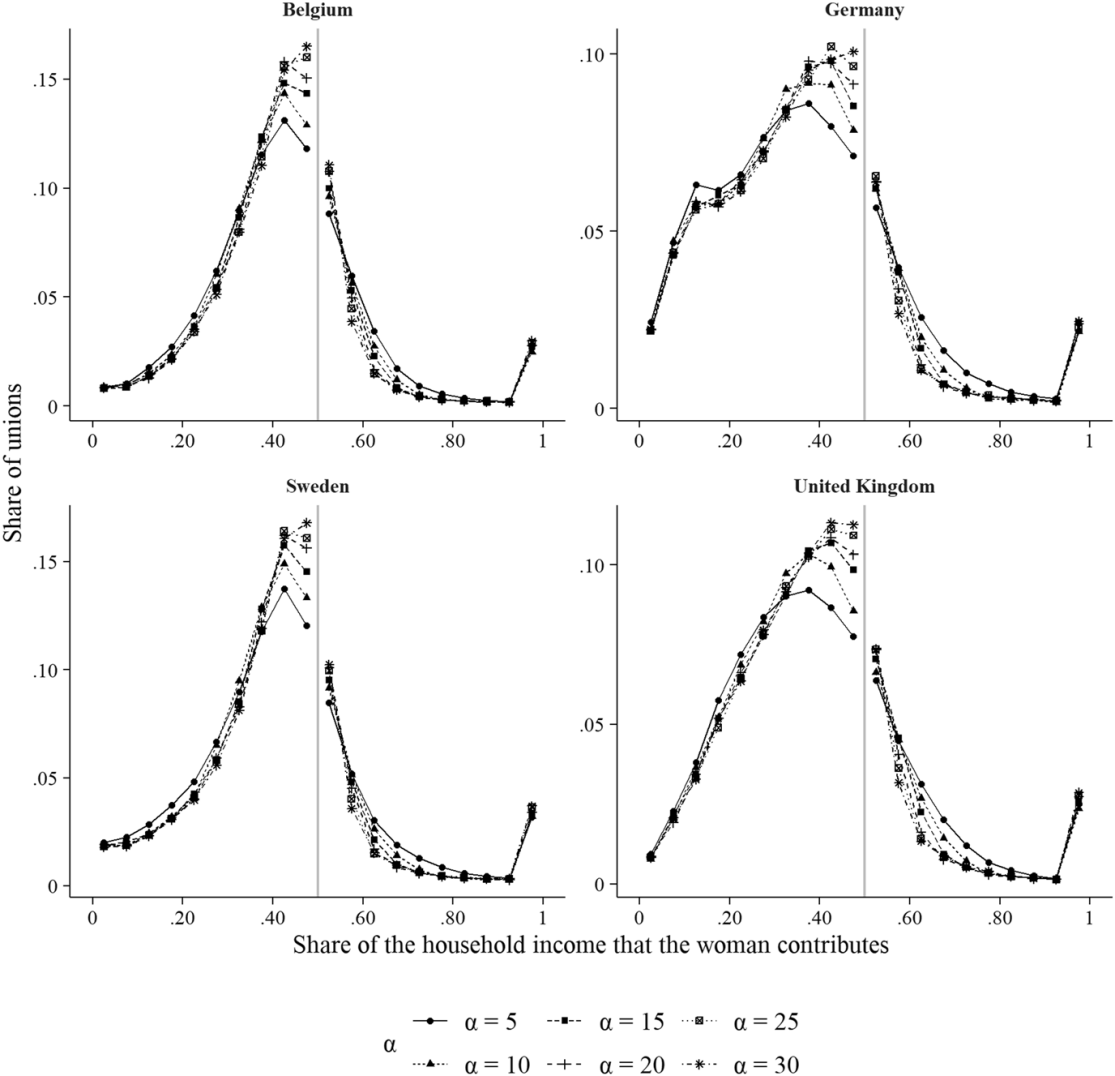
Comparison of the relative income distributions that the simulation model generates, contingent on the parameter *α*. *Note:* The grey vertical line indicates the point where the share of the household income that the woman provides is .5. *Source*: The simulation results are based on the sensitivity simulation experiment

To what extent do our results depend on the assumption that men and women use their own income as a point of reference when selecting a partner? The results of our sensitivity analysis shown in Fig. 5 enable us to answer this question. Remember that the parameter *α* governs how strongly individuals discriminate between potential partners who earn less than they do, and potential partners who earn as much as, or more than, they do. Higher *α*-values imply stronger discrimination. As can be seen in Fig. 5, the cliff in the relative income distribution is the largest for the highest value of *α* (*α* = 30, as we have used in our main experiment). At lower *α*-values, the cliff becomes smaller, and at the lowest *α*-value, the distributions are rather smooth around the 50/50 demarcation line.^6^ Hence, for the cliff to emerge, it is crucial that individuals use their own income as a point of reference when evaluating potential partners.

One remarkable feature of the results shown in Fig. 5 is that as the discontinuity becomes weaker with deceasing *α*-values, the peak of the distribution shifts to the left, so that there often is a large share of couples in which women’s income accounts for about 30–40% of the household income. This provides some insights into precisely how the partnering preferences that we assume here generate the outcomes observed in the main simulation experiment (shown in Fig. 4). The fact that both men and women prefer partners with high income, combined with the fact that women’s average incomes are lower than men’s, leads the relative income distributions to become right-skewed. This means that there are more unions in which women earn considerably less than their partners than there are unions in which they earn more. At the same time, the more strongly members of both genders use their income as a point of reference for selecting partners, the more unions form in which women earn almost as much as their partners, thereby contributing to the cliff at the 50/50 demarcation line. The reason for this is that in this situation, both men and women are aversive of a situation in which their partner makes less than they do. For women it is easier to avoid such a situation, given that men’s average income is higher.

## Discussion and Conclusion

The distribution of the relative contributions of husbands and wives to their joint income shows a remarkable discontinuity at the 50% line: while the number of couples where the wife contributes almost 50% of the household income tends to be quite high in many countries, the number of couples where the wife earns just more than 50% is much lower. It has been suggested that this discontinuity reflects a male breadwinner norm, and more specifically a norm that a man should earn more than his wife. However, in this paper, we have demonstrated that the observed discontinuity in the relative earnings distribution need not reflect such a norm.

We hypothesized that the discontinuity to the disadvantage of women that has been observed empirically in Western countries might emerge even if people would not attach special meaning to a situation in which a wife out-earns her husband and simply value high income in their partners. The reason for this expectation is that there are differences in the average incomes of men and women, and these differences limit men’s opportunities to find partners who earn as much as, or more than, they do, whereas this is easier for women. We have explored the logical consistency and empirical plausibly of our argument with a simple simulation model. The results of our simulation experiments show that this model can generate a discontinuity in the relative income distribution to the disadvantage of women, without the need to assume that there is a norm according to which a man should earn more than his wife. In fact, our results suggest that a discontinuity is likely to emerge even if women and men alike prefer partners with similar or higher income over partners with lower income.

Our work contributes to an emerging body of literature that seeks to shed more light on the mechanisms that might underlie the income cliff, which was first reported for the US by Bertrand et al (2015) (e.g., Binder and Lam 2019; Hederos and Stenberg 2019; Roth and Slotwinski 2018; Sprengholz et al. 2019; Zinovyeva and Tverdostup 2018). In particular, using a similar analytical approach as we did, Binder and Lam (2019) found for the US that if men and women were matched based on their ranks in the respective gender-specific income distribution, existing income differences between the sexes would lead to a right skew (but not necessarily a cliff) in the relative income distribution, even in the absence of a male breadwinner norm. The results that we have presented here for a larger selection of 27 European countries support the notion that existing income differences between men and women can lead to a skew in the relative income distribution to the disadvantage of women. Additionally, we showed that these differences can also create a notable cliff at the 50/50 demarcation line, if both men and women strive for partners whose income is at least as high as their own.

The simulation model that we have presented here is a minimal model that focuses on the mechanism that we were interested in. As such, it abstracts from many additional processes that may affect marriage patterns in real life. For example, one aspect that we have neglected is that peoples’ incomes might be endogenous to the marriage process. There is evidence that especially in more traditional gender-norm contexts, both men and women tend to adjust their labour market behaviour as a result of marriage and the anticipation of children within their newly formed families. Women in particular tend to reduce their working hours to take care of their children (Sanchez and Thomson 1997). This fact may explain some of the deviations that we observed between the empirical data and our simulation results.

To illustrate this, it helps considering the example of Germany, where our model generated considerably fewer couples in which the woman contributes relatively little to the household income than empirically observed. One factor that arguably contributes to this deviation is the German tax-code. In Germany, spouses’ tax burdens are interdependent, so that in marriages in which one partner earns less than the other, there is a financial incentive for the lower earning partner to reduce their participation in the labour force and to reduce their income even further. Given that women’s earning potential tends to be lower than men’s, this usually means that women reduce their working hours and therefore contribute relatively little to the household income (cf. Aboim 2010; Sprengholz et al. 2019). Our model abstracts from such intra-couple income adjustments and therefore fails to match the empirical data in Germany.

Furthermore, we cannot rule out that the income distributions that we have used for initializing our simulation model partly result from gender norms other than a norm that a man should earn more than his wife. As indicated above, women tend to reduce their participation in the labour force more than men upon the birth of a child, and this may partly result from a belief that women are better able to take care of children (Thompson and Walker 1989). Similarly, men are often perceived to be better leaders than women and therefore tend to advance faster through organizational hierarchies and attain higher salaries (Ridgeway 2011). Still, even if such beliefs exist and can explain why there are systematic income differences between men and women, the results of our simulation experiments show that an additional male breadwinner norm at the couple level is not necessary to explain the income inequalities that can been observed within heterosexual unions.

A second factor that we have neglected is other mechanisms that may contribute to similarity in partners’ incomes, net of people’s partner preferences. For example, in our simulations, we have focused on national marriage markets, but in reality, people tend to encounter future partners in more local contexts, such as their schools, neighbourhoods, and workplaces. Such contexts are often socially segregated, and this increases the likelihood that people who meet there are more similar in their socioeconomic characteristics than randomly selected members of the overall population would be (Kalmijn and Flap 2001; Mare 1991; Zinovyeva and Tverdostup 2018). This is likely to increase the similarity in income that can be observed within couples, net of any specific partner preferences. Future research might extend our work to incorporate such more ‘local’ marriage markets in the simulation process and explore how this affects model outcomes.

A third factor that we have neglected are other characteristics that people may consider when selecting a partner, which may be correlated with income. One prime characteristic in this regard is educational attainment. Earlier partner market research has highlighted that educational attainment is a proxy of people’s cultural resources (e.g., their taste in music, political attitudes, etc.), which is one of the most important factors in partner selection, next to people’s socioeconomic resources (as reflected in income) (Grow et al. 2017; Kalmijn 1998). Both men and women tend to prefer partners with similar cultural resources, which leads to high levels of educational homogamy across countries (cf. Blossfeld 2009). At the same time, higher educational attainment is associated with higher income among both men and women, but women tend to earn less than men with the same educational attainment (Bobbitt-Zeher 2007). Thus, to the extent that men and women select educationally similar partners, a cliff in the relative income distribution across households might emerge as a side-effect, even if income itself would play no role in the partner selection process. Future research might consider this possibility and provide intriguing new insights into how much of the cliff might be attributed directly to people’s preferences for socioeconomic resources in their partners, and how much of it might be attributed indirectly to people’s preferences for similar cultural resources in their partners.

A fourth factor that we have not considered is that the amount of information that people have about the composition of the marriage market might vary. In our model, we assumed that people have only minimal information at their disposal and must acquire additional information in a sequential–and subjectively costly–manner. This creates a difficult trade-off between settling for one of the available alternatives and extending the search, without knowing whether better alternatives will present themselves in the future. Some scholars have argued that new digital tools for partner search (such as online dating) have greatly reduced partner search costs, thereby providing people with a more accurate image of ‘who is out there’ (at least within the confines of the chosen dating platform) (e.g., Hitsch et al. 2010). Arguably, assuming such a situation of almost perfect information might have affected our results, by reducing the number of people who settle for a partner who is ‘below’ what would have been possible, given their own attractiveness on the marriage market. We have assessed this possibility with a second simulation model, in which we assumed that (1) men and women know about the income of all the alternatives on the marriage market, and that (2) they can try to date any opposite-sex member at any point in time (with the exception of married individuals and those who have rejected them for a date already). The results of this model generated cliffs in the relative income distributions across countries that were even more pronounced than those reported in this paper. Interestingly, these cliffs occurred already at the point where women provide about 40% of the household income, rather than at the 50% point (results available upon request from the corresponding author). These additional results suggest that our main conclusion does not depend on specific assumptions about the information that people have at their disposal.

Despite the need for further research, our results have important implications for current research practice and for theorizing about the future of gender inequality within families. First, several scholars have highlighted that inferring partner preferences and norms from observed marriage patterns can be an ecological fallacy, because very different preferences can lead to very similar marriage patterns (Binder and Lam 2019; Grow et al. 2017; Grow and Van Bavel 2015; Kalick and Hamilton 1986). Our results add to this body of research and caution against inferring preferences and norms from aggregate-level mating patterns. Note that we have not demonstrated the existence or non-existence of a norm that a husband should earn more than his wife. Rather, we have demonstrated that such a norm is not needed to explain the relative income cliff. Still, such a norm could be one of several mechanisms that can generate a cliff in the relative income distribution. It could well exist, in at least some parts of the population, in at least some countries, and our results suggest that if we want to estimate precisely how much this norm might contribute to observed intra-couples inequalities, we need to take the structure of the marriage market in which unions are formed into account.

Second, several scholars have highlighted that the increase in gender equality that has occurred in Western countries since the 1960s has weakened or even stalled in recent years (e.g., England 2010; Esping-Andersen 2009). One possible reason is that gender norms tend to be deeply entrenched and need time to adjust to structural changes, such as changes in women’s economic roles. Some scholars have therefore suggested that additional gains in equality may occur in the future, to the extent that traditional gender norms fade and become replaced by more egalitarian alternatives (Goldscheider et al. 2015). However, our results suggest that even if people’s partner preferences would be completely gender egalitarian, women may remain economically disadvantaged within their families, if their average income is lower than men’s. Thus, in line with Goldin’s (2014) reasoning, our results suggest that the ‘last chapter of the grand gender convergence’ will not only require ideational change, but also institutional change aimed at reducing the gender pay gap, as this will create the structural conditions that are necessary to attain more equality within families.

## Data Availability

The European Union Statistics on Income and Living Conditions data can be obtained by researchers working in accredited research institutions from Eurostat (for details see https://ec.europa.eu/eurostat/documents/203647/771732/How_to_apply_for_microdata_access.pdf/82d98876-75e5-49f3-950a-d56cec15b896). The scripts of the data analysis and the code of the simulation model can be obtained from https://www.comses.net/codebases/4e97fa4a-db5b-4cff-acea-5314201af5ce/.

## Footnotes

1 Unmarried cohabitation has been on the rise across Europe in recent years and has attained a status similar to marriage in some countries (Hiekel et al. 2014). We thus include both union types in our analysis, but use ‘marriage’ as shorthand in the rest of this paper (for a similar approach see Klesment and Van Bavel 2017).

2 The term ‘two-sex model’ highlights that the model considers the marriage decision of both men and women simultaneously. This is in contrast to ‘one-sex models’, in which only the marriage decisions of men or women are modelled (cf. Willekens 2010).

3 A simplifying assumption is here that individuals prefer having a partner over being single, regardless of income of their partner.

4 Klesment and Van Bavel (2017) only selected women conditional on their own age, regardless of their partner’s age. By contrast, we constrained the ages of both men and women for the following reason. In our simulations, we used the EU-SILC data for separately initializing the income distributions among male and female agents. If for this we would have placed an age constraint on women, but not on men, this would have led to a situation in which income differences between the sexes may partially result from differences in the age distributions in the two samples. To avoid this, we placed the same age constraints on both men and women when generating the input data. As a consequence, we also needed to impose age constraints on both sexes when exploring the empirically observed distributions of relative income within unions. Otherwise, the results of our simulation experiments would be based on very different samples than the result of our empirical analysis.

5 Compared to Fig. 3, we used the observed real incomes in the simulations, without aggregating them into larger income groups.

6 For legibility, we have omitted the share of couples in which women have zero income from the figure.
